# The impact of bilingualism and code-switching on executive function performance

**DOI:** 10.3389/fpsyg.2025.1583441

**Published:** 2025-12-03

**Authors:** Basak Özkara, Raul Schneider, Gülay Cedden, Christiane von Stutterheim, Patric Meyer

**Affiliations:** 1 Institute of Psychology, Heidelberg University, Heidelberg, Germany; 2 School of Psychology, SRH University Heidelberg, Heidelberg, Germany; 3 Institute of German as a Foreign Language Philology, Heidelberg University, Heidelberg, Germany; 4 EEG and Language Processing Laboratory, Faculty of Education, Middle East Technical University, Ankara, Türkiye

**Keywords:** bilingualism, code-switching, executive functions, cognitive control, bilingual advantage

## Abstract

Bilingualism, characterized by the use of two or more languages, places unique demands on executive functions (EFs), which are essential for managing cross-linguistic interference. This study investigates EF performance in Turkish-German bilinguals and German monolinguals across five domains: response inhibiton, working memory, task-switching, interference control, and attention. Additionally, the influence of habitual code-switching behavior on EF outcomes in bilinguals was explored using a novel scene description game designed to elicit naturalistic code-switching patterns, which approximate participants’ habitual bilingual language use. Results revealed that bilinguals outperformed monolinguals in task-switching accuracy, indicating enhanced cognitive flexibility. However, monolinguals exhibited superior working memory performance, as measured by d-prime scores on the N-Back task. No significant group differences were observed in attention-related tasks. Within the bilingual group, the Code-Switching Index (CS Index) emerged as a significant predictor of EF performance, particularly in tasks requiring interference resolution, such as the Stroop Interference test. Higher levels of code-switching were associated with increased susceptibility to Stroop interference, suggesting a complex trade-off between cognitive flexibility and interference control efficiency. These findings contribute to understanding the specific effects of bilingualism on EF performance, highlighting domain-specific adaptations that emerge in some EF components but are absent in others. By integrating insights from EF research with contemporary theories of cognitive control and bilingual language use, this study underscores the importance of analyzing individual EF domains and accounting for diverse bilingual experiences, such as code-switching behavior, to better understand the cognitive mechanisms underlying bilingualism.

## 1 Introduction

Bilingualism, the ability to use two or more languages regularly, characterizes over half of the world’s population (Grosjean, 2021). This linguistic versatility requires bilingual individuals to manage complex cognitive demands, including selecting the appropriate language for a given context and inhibiting interference from the non-target language. Research shows that both languages in a bilingual’s repertoire are simultaneously active during language comprehension and production, even when one language is not overtly used (e.g., Kroll et al., 2006; Hatzidaki et al., 2011). This constant co-activation creates a need for efficient cognitive control mechanisms to manage cross-linguistic interference. These mechanisms are central to the ongoing debate about whether managing multiple languages results in broader cognitive benefits, particularly in executive functions (EFs).

EFs refer to a suite of top-down cognitive processes that enable goal-directed behavior (Miyake et al., 2000; Diamond, 2013). A prominent model by Miyake et al. (2000) proposes three core EF domains: inhibition, shifting, and updating. Inhibition (or *inhibitory control*) refers to the cognitive ability to deliberately suppress dominant, automatic, or prepotent responses when necessary (Miyake et al., 2000). It is typically divided into two distinct subcomponents: interference control (or *interference suppressio*n) and response inhibition. Interference control operates at the attentional level to filter out irrelevant information, while response inhibition operates at the behavioral level to suppress an inappropriate action (Diamond, 2013). Response inhibition is often assessed with the *Go/No-Go* task, where participants respond quickly to target stimuli (Go) but withhold responses to non-targets (No-Go). Interference control is commonly measured using the Stroop (Stroop, 1935), Simon (Hommel, 2011), and Flanker (Eriksen and Eriksen, 1974) tasks, all of which present conflicts between an automatic response and a goal-directed one, requiring suppression of interference to respond correctly. Shifting (or *task-switching*) is the ability to move flexibly between tasks or mental sets as needed (Miyake et al., 2000). It is commonly assessed using task-switching paradigms in which participants alternate between different rules or tasks within a set period. Examples include the Number–Letter task, where participants switch between classifying numbers (odd/even) and letters (vowel/consonant), and the Color–Shape task, which requires categorizing objects by color or shape. Updating is closely linked to working memory and involves the continuous monitoring and quick addition or deletion of information to ensure only the most current and task-relevant information is maintained. It is commonly measured with the N-Back task (Kirchner, 1958), where participants identify when the current stimulus matches one presented *n* steps earlier, with difficulty increasing as *n* rises.

These processes play a critical role in navigating complex, dynamic environments, by allowing individuals to regulate attention, shift between tasks, and suppress irrelevant stimuli. For bilinguals, EFs play a key role in managing the dynamic nature of language use. Inhibition is necessary for avoiding interference from one language while using the other, whereas shifting enables seamless transitions between languages when needed, and updating ensures that language-relevant information is maintained and adjusted as necessary. The *bilingual advantage hypothesis* posits that bilinguals, due to their frequent engagement in cognitive control to manage linguistic competition, develop enhanced EF compared to monolinguals (Bialystok, 2011; Bialystok et al., 2012; Kroll and Bialystok, 2013). Earlier studies largely showed bilinguals outperforming monolinguals on EFs, particularly in the inhibition (Bialystok, 2001; Bialystok et al., 2004, 2012; Colzato et al., 2008) and shifting domains (Garbin et al., 2010; Prior and Gollan, 2011; Prior and MacWhinney, 2010), interpreted as reflecting adaptations that allow for efficient management of two simultaneously active languages. Findings pointing to a bilingual advantage in the updating domain are more limited (Luo et al., 2013; Morales et al., 2013), which may reflect two potentially offsetting effects: experience with dual-language control may enhance bilinguals’ working memory function, while the increased cognitive load of managing two active languages could hinder performance. Taken together, the evidence for a uniform bilingual advantage across EF domains is far from consistent. Consequently, the bilingual advantage hypothesis remains contentious, withmeta-analyses yieldingconflicting results: some report bilingual advantages in EF across age groups (e.g., Grundy, 2020; Yurtsever et al., 2023) while others find no effects or negligible differences between bilinguals and monolinguals (e.g., Lehtonen et al., 2018; Nichols et al., 2020; Lowe et al., 2021; Degirmenci et al., 2022).

A significant limitation of prior studies lies in their tendency to examine only isolated aspects of EF, and often using a single task, rather than assessing multiple components within the same sample. These include studies focusing exclusively on response inhibition (e.g., Kałamała et al., 2020), task switching (e.g., Prior and MacWhinney, 2010; Wiseheart et al., 2016), working memory (e.g., Ratiu and Azuma, 2015), or interference control (e.g., Codere et al., 2013). This approach leaves it unclear how different EF components interact or play out within the same sample, limiting the reliability of comparisons across studies and potentially masking differential effects of bilingualism across EF domains, including effects in opposing directions. While there is an emergence of studies assessing multiple components (e.g., Chen et al., 2025; Antón et al., 2019; Kousaie et al., 2014; Park et al., 2018; Sörman et al., 2019), their numbers remain limited, undermining our ability to draw robust conclusions about bilingualism’s differential effects across domains. In fact, some of these studies have evidenced such trade-offs. For instance, Antón et al. (2019) reported selective bilingual advantages in a demanding (backward repetition) working memory task, despite no group differences in a less demanding (forward repetition) version or in any of the four interference control tasks they used. Kousaie et al. (2014), on the other hand, found no group differences in working memory in either the backward or the forward repetition conditions. Interestingly, they found a bilingual advantage for interference control on a verbal (Stroop) but not on a nonverbal (Simon) task, which the authors proposed might reflect the linguistic nature of the Stroop task. It is debated whether the bilingual advantage is limited to the linguistic domain or transfers to non-linguistic tasks (i.e., near vs. far transfer; Jaeggi et al., 2008, 2014; see Hilchey and Klein, 2011 for a review). More recent studies adopting multi-task approaches likewise reveal mixed results, with selective advantages in some domains but not others (e.g., Chen et al., 2025). These divergent findings can be understood in terms of the persistence–flexibility dilemma (Goschke, 2003) and the dual mechanisms of control framework (Braver, 2012): while proactive control and persistence support stable goal maintenance, reactive control and flexibility enable adaptive shifts. Bilingual language use may shift the balance between these modes of control, enhancing flexibility (e.g., task switching) while reducing persistence in tasks requiring sustained maintenance (e.g., working memory). Such trade-offs underscore the need to examine multiple EF domains within the same sample to capture the specificity of bilingual effects.

A similar drawback arises when distinct processes are collapsed under a general term. For instance, while both response inhibition and interference suppression fall under the domain of inhibition, evidence suggests that bilingual experience can differentially affect these sub-components, highlighting the risk of overgeneralization (Luk et al., 2010; Xia et al., 2022). Addressing these limitations require a more comprehensive approach that evaluates multiple EF domains and sub-components simultaneously, enabling a nuanced understanding of the domain-specific and possibly diverging effects of bilingualism. A growing body of research suggests that the cognitive effects of bilingualism are modulated by variability in bilingual experiences, which may help account for discrepancies in findings. Factors such as age of acquisition (AoA), proficiency levels, and patterns of language use have been identified as important moderators of cognitive outcomes (for a review, see Festman et al., 2023).

One aspect of bilingual experience that has garnered increasing attention is code-switching—the practice of alternating or blending elements from two languages within a single discourse or interaction (Appel and Muysken, 1987). Code-switching is a ubiquitous feature of bilingual communication with considerable variation across individuals in terms of frequency, type, and contextual demands, which can account for variation in cognitive outcomes (see Beatty-Martínez et al., 2020; Beatty-Martínez and Dussias, 2017). In his seminal work on bilingualism, Muysken (1997, 2000, 2013) distinguishes between three types of intra-sentential code-switching which differ in the degree of co-activation of the two languages: alternation, insertion, and dense code-switching (or *congruent lexicalization*) (Table 1). Accordingly, alternation refers to a complete switch including grammar and lexicon, and requires relatively low co-activation of the two languages. Insertion involves integrating a lexical item from one language into the structure of the other (i.e., matrix language), thus requiring lexical-level co-activation. Dense code-switching on the other hand refers to the integration of both languages within a clause andwith no clear matrix language, therefore requiring high co-activation at both lexical and grammatical levels.

**Table 1:** Muysken (2000)‘s code-switching types as observed in naturalistic speech of Turkish-German bilinguals, German marked in bold (Treffers-Daller, 2020).

CS type	Example
Insertion	Bütün **Flughafen**’ı bul-du-m. *Entire **airport-**ACC. found-PAST-1. SG.* “I found the entire airport.”
Alternation	On-dan sonra balo-ya git-tiğ-imiz-de **sind wir telephonieren gegangen.** *That-ABL. after balo-DAT. go-FNOM.-1. PL-LOC. **are we telephone gone.*** “After that, when we went to the ball, **we went out to give a call.”**
Dense CS	Und ben **feiern** yap-a-ma-dı-m, çünkü **an dem Tag wo Klassenfahrt**’a gid-ecek-ti-m, akşam-a **konnt’ keine Fete machen.** *And I **party** do-ABIL.-NEG.-PAST-1. SG because **on the-DAT. day where school trip**-DAT. go-FUT.-PAST-1. SG evening-DAT. **could no party make.*** “And I could not go to the party because on the day that I was going to the school trip I could not have a party until the evening.”

From a cognitive perspective, code-switching is not merely a linguistic phenomenon but a dynamic behavior that engages diverse control processes. Frameworks such as the Adaptive Control Hypothesis (ACH) (Green and Abutalebi, 2013) and the Control Process Model (CPM) (Green and Wei, 2014) formalize these links and provide theoretical insights into how different types of code-switching and interactional contexts modulate cognitive demands. For example, the ACH categorizes bilingual language use into single-language, dual-language, and dense code-switching contexts, each with distinct demands on cognitive control processes like goal maintenance, conflict monitoring, and interference suppression. In a dense code-switching context, where both languages are actively blended, cognitive demands may shift from competitive control to cooperative strategies, reducing the need for interference control (Green and Abutalebi, 2013; Green, 2018). Similarly, the CPM differentiates types of code-switching based on Muysken (2000) and proposes unique modes of control engaged by each typebased on the level of inhibition involved (Green and Wei, 2014). More recently, the dual mechanisms of control (Braver, 2012) has emerged as a prominent approach for examining the influence of code-switching on EFs (e.g., Beatty-Martínez et al., 2020; Hofweber et al., 2020; Jiang et al., 2023; for a review, see Özkara et al., 2025).

Most studies investigating the influence of code-switching and EF date have treated code-switching as a single construct, overlooking distinctions between different code-switching types. Overall, existing research produced mixed results, potentially due to methodological challenges, including inconsistencies in defining, operationalizing and measuring code-switching behavior (Özkara et al., 2025). In particular, many studies rely on self-reported measures of language use, which may fail to capture the dynamic and context-dependent nature of code-switching in everyday life (Cedden et al., 2024). Major limitations of self-reports of code-switching include the cognitively complex and often unconscious nature of the behavior, the high metalinguistic awareness required to accurately identify and differentiate types of code-switching, susceptibility to recall errors, and the influence of social stigma and attitudes, all of which undermine the accuracy and validity of the data (Hofweber et al., 2019). Two commonly used self-report measures of code-switching are acceptability and frequency judgment tasks, in which participants rate the acceptability of presented code-switched structures and the frequency with which they encounter or produce specific types of code-switching, respectively. Besides their reliance on metalinguistic awareness, these measures may also be susceptible to individual attitudes toward code-switching (Badiola et al., 2018). Several alternative approaches have been proposed to circumvent reliance on self-report data, and ultimately improve our understanding of how bilinguals’ real-time, uncued language-switching behaviors influence EF performance. These include novel tasks designed to elicit language production such as the bilingual email production task (Hofweber et al., 2019), code-switching map task (Beatty-Martínez and Dussias, 2017), and referential communication task (Valdés Kroff and Fernández-Duque, 2017).

To address these limitations, the present study investigates the cognitive implications of bilingualism by focusing on two interconnected research questions:

RQ1: Does bilingualism lead to measurable adaptations in EF performance, and do these effects vary across different EF domains?
RQ2: Does bilinguals’ habitual code-switching behavior, approximated via patterns elicited in real time, predict their EF performance, and are these effects domain-specific?

To address RQ1, we compare Turkish-German bilinguals and German monolinguals across five EF domains: response inhibition, working memory, task-switching, interference control, and attention. We assess response inhibition through a Go/No-Go task in order to capture selective response inhibition, a common control process implicated in bilingual language production that halts an ongoing response to allow a more task-appropriate one, such as when a new conversational partner arrives (Green and Abutalebi, 2013). Working memory is assessed using an N-Back task, for a direct assessment of updating processes where evidence for bilingual effects remain limited and inconclusive. Task-switching is assessed using an alternating-runs paradigm as a measure of the ability to efficiently shift between tasks or mental sets, a process frequently engaged in bilingual language control. For interference control, a verbal Stroop task is used instead of nonverbal paradigms to reflect the language-based conflict bilinguals experience in suppressing competing lexical representations, noting that any observed effects could be limited to the linguistic domain. Finally, attention is measured using intrinsic alertness and divided attention tasks, serving as a baseline function that supports the other EF domains and helps isolate their specific contributions.

To address RQ2, we use a novel, interactive scene description game that simulates real-life communication with a bilingual confederate. By allowing participants to code-switch naturally, as they would in everyday interactions, the task is designed to overcome the limitations of self-reported data and yield a detailed, ecologically valid estimate of habitual code-switching behavior. The resulting code-switching profile is then analyzed to examine how individual differences in these patterns predict performance across EF domains.

We predict significant differences between bilinguals and monolinguals in EF performance, with bilinguals demonstrating advantages in specific domains. However, these advantages are expected to vary across EF domains, reflecting domain-specific effects of bilingualism. Specifically, we expect bilinguals to outperform monolinguals in tasks assessing response inhibition, task-switching and interference control due to their frequent practice in managing competing linguistic systems. For working memory, we predict no group differences or relative disadvantages for bilinguals, particularly due to the expected cognitive load associated with their exposure to and use of code-switching in the preceding scene description game. Similarly, we expect no group differences in the attention domain, based on the assumption that basic attentional functions are not directly influenced by bilingualism unless they are required for tasks that also recruit other EF domains. Furthermore, we hypothesize that within the bilingual group, code-switching behavior will predict EF performance. Specifically, we expect that a higher proportion of dense code-switching (i.e., high co-activation) relative to alternations and single-word insertions (i.e., low co-activation) would predict enhanced task-switching performance, while imposing costs on interference control efficiency.

By integrating insights from the bilingual advantage literature with contemporary theories of cognitive control, this research aims to contribute to the ongoing dialogue on the cognitive benefits of bilingualism. Specifically, it seeks to elucidate the extent to which bilingual experiences, particularly habitual code-switching, shape EF performance. The findings are expected to provide new perspectives on the cognitive and linguistic mechanisms underlying bilingualism and address important questions in the bilingual advantage debate.

## 2 Materials and methods

### 2.1 Participants

Participants were young adults aged 18–30 recruited as part of a larger project on Turkish-German bilingualism funded by the German Research Foundation (DFG) and paid 10 euros per hour for their participation. The preliminary sample consisted of 72 participants. 6 participants (5 bilingual, 1 monolingual) were excluded due to self-reported attention related disorders, resulting in a final sample of 66: 31 Turkish-German bilinguals (23 female) and 35 German monolinguals (21 female, 3 diverse). All participants except one were born in Germany. A language background questionnaire was administered to all participants to characterize their language history and confirm eligibility for the study. All bilingual participants were either simultaneous (*N* = 13), or early sequential bilinguals who reported acquiring the German language prior to the age of 7 and receiving their formal schooling in Germany. The monolingual group was composed of participants who did not have active or sustained exposure to a second language before formal schooling and who had not lived in or spent significant time in a non-German-speaking country. Importantly, local dialects of German (e.g., regional varieties) were not classified as a second language, as they share high structural and lexical overlap with Standard German and are typically acquired as intralinguistic variants rather than distinct linguistic systems (Auer, 2005; Trudgill, 2019). While bidialectalism has been suggested to engage control processes in ways partially comparable to bilingualism (Kirk et al., 2014; Antoniou et al., 2016), its cognitive effects are generally reported to be weaker and less consistent. Therefore, although the presence of dialect knowledge cannot be entirely ruled out, we consider its influence on the present group classification to be limited. Similarly, while most participants had some knowledge of English through formal schooling, this was not considered equivalent to early and sustained naturalistic bilingualism. The bilingual and monolingual groups were matched on age and highest level of education achieved to minimize potential confounding effects on EF performance.

To further characterize bilinguals’ language experience, participants completed a language usage questionnaire assessing the relative use of German and Turkish across various domains of daily life, including the home, workplace or study environment, leisure activities, and passive language exposure. The design of this questionnaire was informed by the concept of language entropy proposed by Gullifer and Titone (2020). Based on participants’ responses, language entropy scores were calculated to estimate the relative likelihood of each language being used in a given context and to capture the overall diversity of their language practices (Table 2). Scores ranged from 0 to 1, with higher values reflecting more balanced and less predictable language use across contexts, and lower values indicating more context-specific, single-language use.

**Table 2:** Mean language entropy scores for bilingual participants across different contexts of language use.

Context of language use	Mean entropy score (S. D.)
Home	0.94 (0.10)
Place of work/study	0.59 (0.31)
Leisure	0.88 (0.23)
Passive language consumption	0.83 (0.27)
Overall	0.87 (0.18)

Higher values indicate more balanced use of German and Turkish and lower predictability of which language will be used in a given context.

### 2.2 Materials

#### 2.2.1 EF tasks

The study utilized a comprehensive battery of cognitive tasks administered via the SCHUHFRIED Vienna Test System (VTS; Schuhfried GmbH, 2013). Each task was designed to evaluate specific domains of EFs, leveraging established paradigms with demonstrated validity and reliability. As trial-level data were not available for our sample, we refer to the reliability values reported in the official manuals for each task listed below. The specific EF tasks used, and their respective domains are outlined in Table 3.

- Response Inhibition (Go/No-Go Task, INHIB; Kaiser et al., 2024). The INHIB test was used to assess the ability to suppress automatic or inappropriate responses using a go/no-go paradigm. Participants were asked to respond to frequent stimuli (e.g., triangles) and inhibit responses to rare stimuli (e.g., circles). Stimuli were presented for 200 milliseconds (ms), with inter-stimulus intervals of 1 s. The task included 125 trials, comprising 101 frequent and 24 rare stimuli presented in a single block. Outcomes included the sensitivity index (d’), reaction times, and errors (commission and omission). The administration time for the test was approximately 4 min.
- Working Memory (N-Back Task, NBN; Schellig et al., 2011). The NBN task was used to evaluate working memory capacity through a 2-back nonverbal paradigm using stimuli that minimize verbal mediation. Participants observed a sequence of 100 abstract figures, each displayed for 1,500 milliseconds (ms) with an inter-stimulus interval of 1,500 ms, presented in a single block. They were required to identify when the current figure matched the one presented two trials earlier. The administration time for the test was approximately 9 min.
- Interference Control (Stroop Task, STROOP; Schuhfried, 2024). The Stroop task was utilized to measure the ability to manage conflicting information, operationalizing the ability to suppress automated responses in favor of less habitual ones. In the version used for this study, a color-word (e.g., “red,” “green,” “yellow,” or “blue”; always presented in German) appeared in the upper third of the screen, written in one of these four colors. Four corresponding color buttons were displayed at the bottom of the screen, aligned with the participant’s keyboard. The task consisted of two parts. In the first part, participants were instructed to respond based on the semantic meaning of the color-word, disregarding the color in which it is written. In the second part, participants responded based on the color of the text while ignoring the word’s semantic meaning. Each part included both congruent items (i.e., YELLOW written in yellow) or incongruent items (i.e., YELLOW written in red). The next item was presented immediately after the participant’s response, maintaining a continuous and fast-paced task flow. The administration time for the test was approximately 10 min.
- Task-Switching (SWITCH; Gmehlin et al., 2018). The Task Switching task was employed to assess cognitive flexibility, requiring participants to alternate between two tasks based on stimulus properties shape (triangle/circle) and brightness (gray/black). Stimuli were presented in a predictable alternating-runs paradigm in a single block, switching tasks every two trials (e.g., AA BB). Participants were asked to press one of two buttons to respond, with congruent stimuli (e.g., a light gray triangle) requiring the same button for both tasks, and incongruent stimuli (e.g., a dark gray triangle) requiring different buttons depending on the task. The assignment of stimulus attributes (form/brightness) to motor reactions (button presses) was arbitrary and had to be learned during the practice phase. In the test phase, a target stimulus appeared centrally on the screen, and participants had a response window of 5,000 ms to react. Once a correct response was entered the stimulus disappeared, and a new one followed an interval of 750 ms. Reaction times and error rates were recorded for both switch trials (where participants switched between tasks) and repeat trials (where they repeated the same task), allowing for the calculation of switch costs as a measure of task reconfiguration and interference suppression. The administration time for the test was approximately 12 min.
- Attention (WAF; Sturm, 2024). WAF perception and attention functions battery was utilized to evaluate two aspects of attentional control: intrinsic alertness and divided attention. Two consecutive tasks assessed participants’ ability to maintain focus and allocate attention effectively across sensory modalities, addressing both the intensity and selectivity dimensions of attention. The Intrinsic Alertness Task (visual) was used capture the participant’s intrinsic alertness, representing their ability to maintain baseline attentional readiness. Participant were required to respond as quickly as possible to the appearance of simple visual stimuli—black circles presented unpredictably on the screen. Each signal was displayed for 1,500 ms before disappearing with inter-stimulus intervals varying between 3 and 5 s. Reaction times were recorded as the primary outcome measure. The subtest consisted of 25 presented in a single block stimuli and had a total administration time of approximately 2 min, offering a concise evaluation of attentional intensity. The Cross-modal Divided Attention Task (visual/auditory) assessed the participant’s ability to manage attentional resources effectively across simultaneous sensory channels Participants monitored stimuli that can be either relevant (visual: square; auditory: high-pitched tone) or irrelevant (visual: triangle; auditory: low-pitched tone) presented in a single block. They were instructed to respond only when a relevant stimulus appeared consecutively. Each stimulus was presented for 1,500 ms with an interstimulus interval of 1,000 ms. The subtest included 85 stimuli, 21 of which were relevant, and the administration time was approximately 6 min.

**Table 3:** EF tasks and their corresponding domains.

EF domain	EF task used
Response inhibition	Go/No-Go task
Working memory	N-Back task
Interference control	STROOP Interference test
Task switching	Task-switching task
Attention	Intrinsic Alertness Task Cross-Modal Divided Attention Task

#### 2.2.2 Scene description game

A scripted scene description game (code-switching game; CS game) was developed in order to elicit and assess bilinguals’ habitual code-switching patterns in a controlled yet ecologically valid setting (Dieck et al., 2025, Submitted Manuscript)^1^. As part of a larger research project on bilingualism, this game was the first task participants engaged in upon arrival. Administering the game before any other tasks, including demographic questionnaires, allowed us to capture the participants’ code-switching behavior as close to its authentic form as possible, as participants would inevitably become more aware of the objectives of the study regarding their language use. In the game, bilingual participants engaged in a reciprocal scene description task with a bilingual confederate, who was introduced to them as another participant. The confederate followed a scripted set of descriptions, which was identical for all participants to ensure consistency. Some scenes were described entirely in German, while others were described exclusively in Turkish. Most scene descriptions, however, incorporated both languages, reflecting common code-switching patterns observed in Turkish-German bilinguals, such as insertions, alternations, and congruent lexicalization to varying degrees (for examples, see Table 4). These patterns were informed by existing corpus-based research on Turkish-German bilingual communities (Treffers-Daller, 2020), and were designed to resemble the linguistic variability found in natural bilingual interactions. The expectation was that participants would use their languages in a way that mirrored their everyday bilingual interactions, influenced by the linguistic behavior of the confederate.

**Table 4:** Examples of the confederate’s scripted descriptions in the scene description game, illustrating code-switching patterns with Turkish and German as matrix languages and alternation without a clear matrix language.

Matrix language	Confederate script example
Turkish	**Bir çocuk** Fußball spielen **yaparken top cama gelmiş** und **cam kırılmış. Evde oturan nine de herhalde çocuğu** erwischen **yapmış ve çocuğa kızıyor. Nine çok kızgın gözüküyor.** *(It seems that while a child was playing football, the ball hit the window and broke it. The grandmother who lived in the house apparently caught the child and is scolding him. The grandmother looks very angry.)*
German	Zwei Kinder sind mit ihrer Oma in der Küche und machen zusammen **köfte**. Auf einem Teller liegen schon einige **köfte**s. Der Junge hilft seiner Oma und schüttet Mehl nach **ve kız da havuç** schneiden **yapıyor**. Sie macht glaube ich einen Salat oder eine Suppe. *(Two children are in the kitchen with their grandmother, making meatballs together. There are already some meatballs on a plate. The boy is helping his grandmother and pouring in flour, and the girl is cutting carrots. I think she’s making a salad or a soup.)*
Alternation	**Kız merdivenle ağaca çıkmış ve meyve topluyor. Bir kız ve bir oğlan da merdiveni tutuyorlar** damit das Mädchen nicht runterfällt. **Çoktan bir kaç meyve toplayıp sepete koymuşlar. Bir kaç meyve de yere düşmüş.** Das sind glaube ich Äpfel. *(It seems that the girl climbed up the tree with a ladder and is picking fruit. A girl and a boy are holding the ladder so that the girl does not fall down. They’ve already picked some fruit and put it in the basket. A few fruits have also fallen to the ground. I think those are apples.)*

Turkish in bold, translations in parentheses.

### 2.3 Procedure

#### 2.3.1 EF tasks

The administration of EF tests was structured into two sequential blocks administered on two separate days, designed to optimize participant engagement and minimize fatigue. The tasks in each block were administered in a fixed order for all participants, with the sequence designed to balance cognitive demands and ensure an efficient flow, and result in blocks of similar duration. The first block included the Go/No-Go task, N-Back task, and the Stroop Interference test, presented in this fixed sequence. The second block consisted of the Task-Switching task, followed by the Intrinsic Alertness and Cross-Model Divided Attention tasks. Before starting each task, participants completed a brief practice session to familiarize themselves with the task requirements and ensure comprehension of the instructions. Practice sessions included a small number of trials representative of the actual task conditions, allowing participants to adjust to the response format and task demands without affecting their performance during the actual testing phase. All task instructions and on-screen prompts were presented in German, ensuring consistency and accessibility for both monolingual and bilingual participant groups. This uniform presentation minimized the influence of language comprehension on task performance. Testing took place in a controlled laboratory setting with minimal distractions, and participants were encouraged to ask clarifying questions before starting each task to ensure a clear understanding of the procedures.

#### 2.3.2 Scene description game

For the scene description game, the bilingual participant was welcomed into the experimental room and seated across from a confederate, each in front of a computer screen. The confederate was carefully presented as another participant to create a peer-like interaction. Throughout the session, the participant and the confederate were addressed collectively to foster the impression that the activity was new to both, reducing any sense of formality and encouraging natural communication. The players were informed that they were both Turkish-German bilinguals and could use either or both languages freely, as they would in everyday interactions with other bilinguals. This setup was reinforced by the confederate’s role as an in-group member, a crucial factor in promoting naturalistic code-switching behavior, as evidenced by prior research (Auer, 1984; Kootstra and Muysken, 2017). Instructions for the game were delivered verbally in Turkish by an experimenter who was a monolingual Turkish speaker, further activating the bilingual participant’s Turkish language mode. The experimenter emphasized that the participants were free to communicate in whichever language they preferred and that there were no restrictions on language use during the game. Participants were informed that the session would be audio-recorded to capture their language behavior for subsequent analysis. The game began once the experimenter had left the room, allowing the participants to interact without external monitoring. During each trial, the describing player detailed an image displayed on their screen, while the player receiving the description viewed two images: one accurately matching the description and another serving as a distractor. The receiving player selected the correct image via a mouse click, at which point the roles reversed. This alternation continued for 10 rounds, resulting in a session lasting approximately 10 min. The confederate adhered to the scripted set of descriptions for her turns, ensuring consistency across all participants. During the participant’s turns, the confederate encouraged detailed scene descriptions by actively engaging, asking clarifying questions when appropriate, and responding naturally to maintain the conversational flow. This interaction aimed to create an environment conducive to eliciting the participant’s habitual code-switching behavior.

### 2.4 Statistical analyses

All statistical analysis were conducted to examine the relationships between bilingualism, EF performance, and code-switching behavior, as well as to explore potential group differences between bilingual and monolingual participants. Data preprocessing, descriptive analyses, inferential tests, and regression modeling were performed using RStudio (v.2024.04.1 + 748 and 2024.09.0 + 375) and JASP (v.0.19.3). Statistical significance was evaluated at an alpha level of *p* < 0.05, with corrections applied for multiple comparisons as necessary. Effect sizes were reported alongside *p*-values to provide additional context for interpreting the results. Data preprocessing included the identification and handling of missing data, outliers, and violations of statistical assumptions. Outliers were defined as values exceeding two standard deviations from the mean and were excluded on a case-by-case basis. The normality of continuous variables was assessed using the Shapiro–Wilk test and Q-Q plots. Homogeneity of variances across groups was tested using Levene’s test. Non-normally distributed variables were log-transformed where appropriate to meet the assumptions of parametric tests. Descriptive statistics were calculated for all variables, including EF task performance, CS Index scores, and demographic variables such as age, gender, and education level. Moreover, any findings indicating a trade-off effect in EF performance were additionally analyzed with an ANCOVA.

#### 2.4.1 Group analyses

To compare EF performance between bilinguals and monolinguals, independent samples t-tests were conducted for variables meeting the assumption of equal variances, as determined by Levene’s test. For variables where the assumption of equal variances was violated, Welch’s t-tests were applied to provide a more robust comparison. These tests were conducted separately for each EF domain, enabling a detailed examination of potential differences in performance across groups. For non-normally distributed variables, Mann–Whitney U tests were used as a non-parametric alternative. Effect sizes were reported alongside *p*-values to provide context for interpreting the practical significance of findings. Cohen’s 𝑑 was calculated for independent samples *t*-tests and Welch’s t-tests, while Rank-Biserial Correlation was used for Mann–Whitney *U* tests.

Given the number of statistical tests performed, the potential for Type I errors was addressed using false discovery rate (FDR) corrections for exploratory analyses and Bonferroni adjustments for confirmatory analyses. Results were interpreted with an emphasis on effect sizes and confidence intervals to mitigate overreliance on p-values. The specific outcome variables included in the analyses are summarized in Table 5.

**Table 5:** Specific EF outcome variables used for statistical analyses.

EF task	Outcome variables	Description
Go/No-Go Task	Sensitivity Index	Measure of overall performance based on number of false alarms subtracted from correctly processed “go” stimuli (i.e., hits)
	Reaction Time	Mean reaction time for hits
N-Back Task	Number of correct trials	
	Number of error trials	
	Number of omission trials	
	Reaction Time Correct Trials	Mean reaction time for hits
	Reaction Time Errors	Mean reaction time for errors
	d-prime	Number of errors subtracted from number of correct responses
STROOP Task	Reaction Time Reading (Congruent)	Median of reaction time for reading congruent trials
	Reaction Time Reading (Incongruent)	Median of reaction time for reading incongruent trials
	Reaction Time Naming (Congruent)	Median of reaction time for naming congruent trials
	Reaction Time Naming (Incongruent)	Median of reaction time for naming incongruent trials
	Reading Interference Tendency	Calculated as median of reaction times for reading incongruent stimuli minus median of reaction times for reading congruent stimuli
	Naming Interference Tendency	Calculated as median of reaction times for naming incongruent stimuli minus median of reaction times for naming congruent stimuli
Task-Switching Task	Accuracy	Local switching costs for the processing errors, i.e., the difference between the percentage of correct responses for switching and repeated tasks.
	Speed	Local switching costs for the processing time, i.e., the difference between the mean reaction times for switching and repeated tasks.
Intrinsic Alertness Task	Intrinsic alertness (visual)	Logarithmic mean of the individual reaction times
Cross-Modal Divided Attention Task	Cross-modal divided attention (visual/auditory)	Logarithmic mean of the individual reaction times

#### 2.4.2 Bilingual within-subject analyses

The recordings from the scene description game were transcribed by trained bilingual research assistants. In order to obtain a quantitative measure of code-switching, we used a novel method developed by Dieck et al. (see footnote 1) as part of the same project. Transcriptions were coded using a detailed framework that categorized different types of code-switching behavior, such as single-word insertions, alternations between languages, and dense code-switching (Muysken, 2000, 2013). Additional subcategories specific to Turkish-German CS (morphological insertions; yapmak/etmek-constructions) were also coded. These data were used to compute a comprehensive code-switching index (CS Index or “switchindex”) for each participant. Per participant, code-switching categories were grouped and summarized, and proportions calculated by dividing the number of code-switching tokens by total utterances. Proportions of alternation switching and single-lexeme insertions (low co-activation) were calculated separately from dense switching phenomena (high co-activation). This resulted in two proportions per participant. These were plotted in a two-dimensional space, and the Euclidean distance from the origin (0,0) was used as a unidimensional measure of overall code-switching behavior, yielding a continuous numeric value referred to as the “switchindex” (for more details, see footnote 1).

To investigate the predictive role of the CS Index on EF performance within the bilingual group, multiple linear regression models were employed. Before conducting regression analyses, a correlation matrix was computed for all measured variables, including EF task performance, CS Index and demographics. Only variables that showed significant or near-significant correlations (*p* < 0.10) with the dependent variables of interest were selected for further multiple linear regression analyses. EF outcome variables served as dependent variables, while the CS Index, age of acquisition (AoA), and level of education were included as independent predictors. The CS Index, calculated based on transcribed data from the scene description game, was treated as a continuous variable. Model fit was evaluated using *R^2^* and adjusted *R^2^* Standardized regression coefficients (*β*) were reported for all predictors to facilitate interpretation of effect sizes. Alpha error correction was applied to account for multiple comparisons and to reduce the likelihood of Type I errors in all calculations. The multiple linear regression models were specified using a sequential approach. First, models including the EF outcome variables were constructed. Second, more comprehensive models including both the EF outcome variables and demographic variables were specified. Within each of these model specifications, predictors were selected using a backward elimination method. All variables for a given model were initially entered simultaneously, and non-significant predictors were then systematically removed to arrive at the most parsimonious and powerful model. Following variable selection, the final models were assessed for multicollinearity. The Variance Inflation Factor (VIF) for all retained predictors was well below the standard threshold of 5, confirming that multicollinearity was not a concern for the interpretation of the results.

## 3 Results

### 3.1 Group analyses

#### 3.1.1 Demographics

Descriptive statistics for demographic and linguistic variables are summarized in Table 6. The bilingual group and monolingual group were matched in terms of age (𝑀bilingual = 24.4, 𝑆𝐷=3.7; 𝑀monolingual = 25.9, 𝑆𝐷 = 3.2) and education level (𝑀bilingual = 4.7, 𝑆𝐷 = 0.7; 𝑀monolingual = 4.8, 𝑆𝐷 = 0.6). The groups did not significantly differ in gender distribution (*χ^2^* (1) = 0.49, *p* = 0.485). Within the bilingual group, the mean age of acquisition (AoA) for German was 2.1 years (*SD* = 1.7), with 41.2% of participants acquiring German before the age of 3.

**Table 6:** Summary of participants’ demographic data.

Statistic	Bilinguals		Monolinguals	
	Mean	Range	Mean	Range
Age	24.4	18–34	25.9	21–33
Education level*	4.7	2–5	4.8	2–5
Bilingual AoA**	2.1	0–6		

^*^Education level was assessed using a 5-point Likert scale based on the German school system, where 1 indicates the lowest level attained and 5 the highest.

^*^Bilingual AoA refers to the average age of acquisition of the German language in the bilingual group.

#### 3.1.2 EF performance

##### 3.1.2.1 Response inhibition

**Figure 1:**
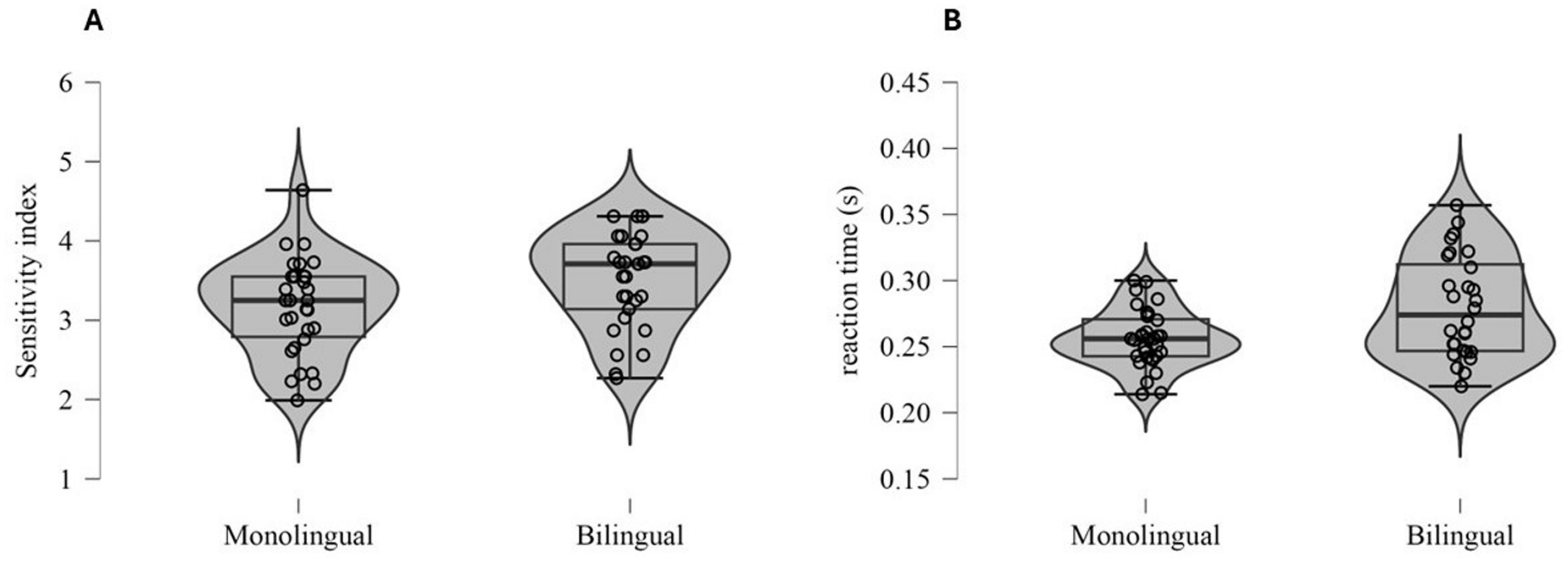
Sensitivity index **(A)** and reaction time **(B)** on the Go/No-Go task for monolingual and bilingual participants.

Bilingual participants outperformed monolinguals in the Go/No-Go task as reflected in their significantly higher *sensitivity index* scores (*t* (57) = −2.12, *p* = 0.039, Cohen’s *d* = −0.55). However, bilinguals exhibited slower reaction times compared to monolinguals (*t* (41.68) = −2.93, *p* = 0.006, Cohen’s *d* = −0.77) (Figure 1).

When performance was controlled for reaction time, the group difference in sensitivity index scores was no longer significant (*F* (1, 55) = 1.836, *p* = 0.181, η^2^ = 0.030). In contrast, a significant difference in reaction time remained, with bilinguals showing slower reaction times than monolinguals, even after controlling for sensitivity index scores (*F* (1, 55) = 5.739, *p* = 0.020, η^2^ = 0.089).

##### 3.1.2.2 Working memory

**Figure 2:**
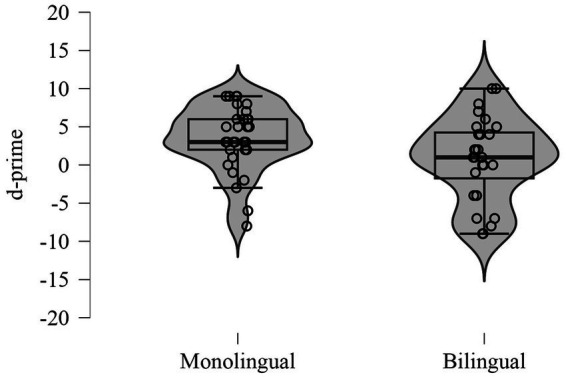
Performance on the N-back task for monolingual and bilingual participants.

Monolinguals outperformed bilinguals on the N-back task, as evidenced by significantly higher d-prime scores (*U* = 588.00, *p* = 0.038, Rank-Biserial Correlation = 0.313) (Figure 2), suggesting an advantage for monolinguals in working memory accuracy. No significant differences were observed in other measures of the N-Back task.

##### 3.1.2.3 Task-switching

**Figure 3:**
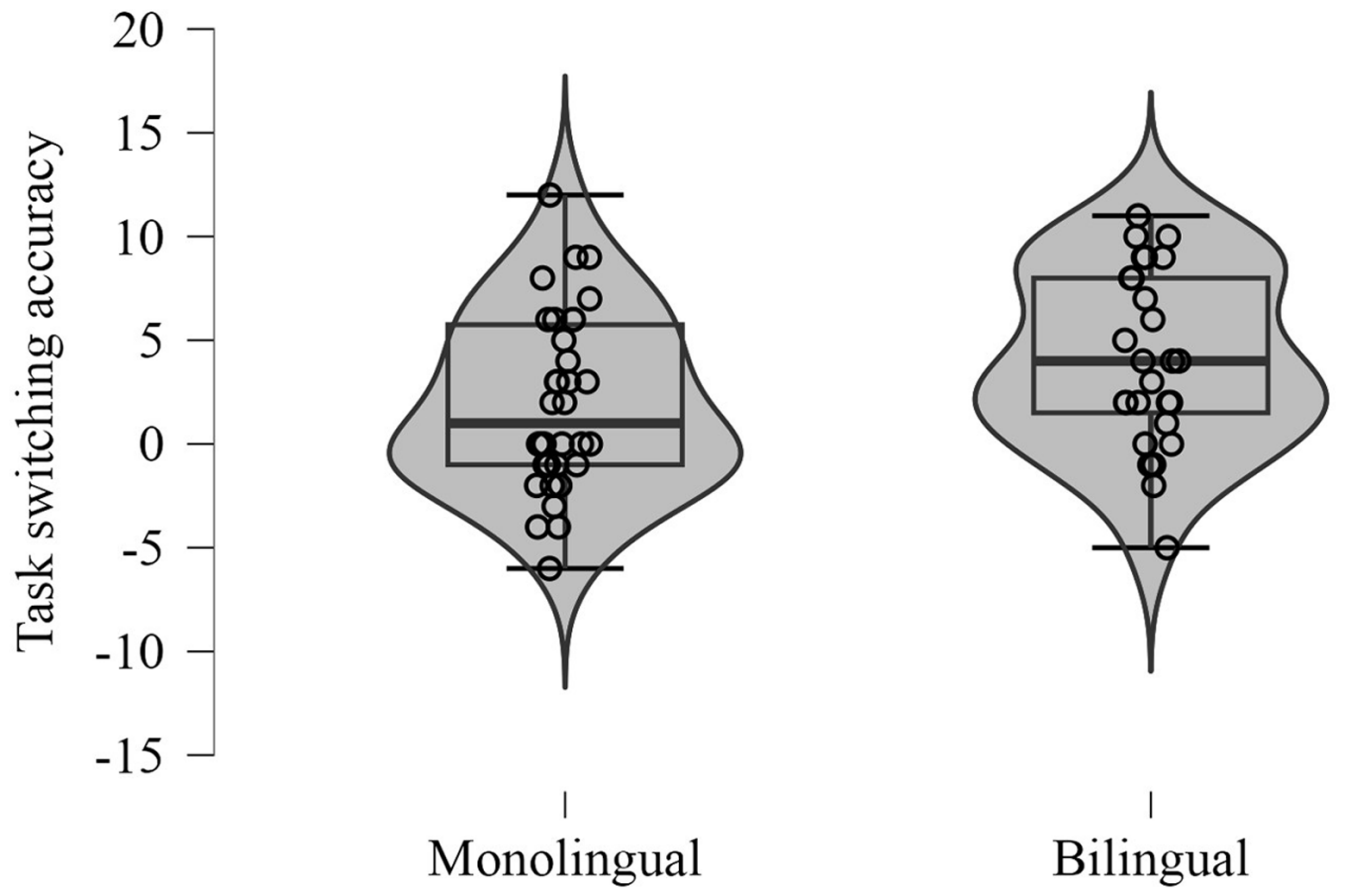
Task-switching accuracy scores for bilingual and monolingual participants.

Bilinguals demonstrated significantly higher accuracy in the Task-switching task (Student t-Test: *t* (60) = −2.00, *p* = 0.05, Cohen’s *d* = −0.51) (Figure 3). There were no significant differences in task-switching speed (*p* = 0.629).

##### 3.1.2.4 Interference control

**Figure 4:**
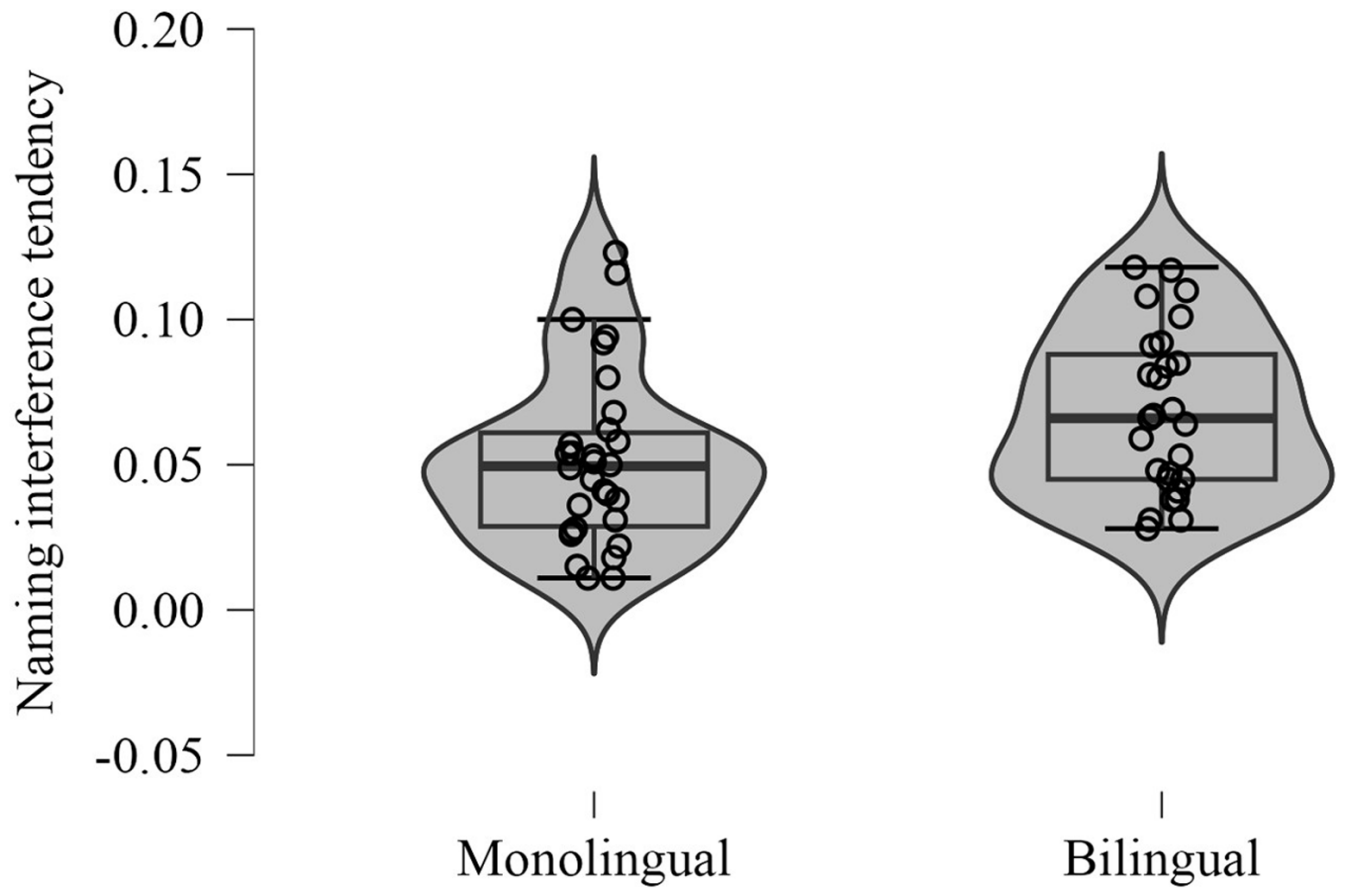
Naming interference tendency scores on the Stroop task for bilingual and monolingual participants.

In the Stroop Interference test, bilinguals showed significantly higher *naming interference tendency* scores (*U* = 293.50, *p* = 0.05, Rank-Biserial Correlation = −0.30) (Figure 4), suggesting greater susceptibility to interference from incongruent stimuli. However, no significant differences were found in other measures of the Stroop task.

##### 3.1.2.5 Attention

No significant group differences were observed in the Intrinsic Alertness task (*p* = 0.60) or the Cross-Modal Divided Attention Task (*p* = 0.73), as assessed by the WAF battery, indicating no specific bilingual effects in attention-related tasks under the current experimental conditions. Means and standard deviations for each group and EF outcome variable are reported in the Supplementary material.

### 3.2 Bilingual within-subject analyses

The analysis revealed a significant positive correlation between the CS Index and Stroop reading interference tendency (*r* = 0.476, *p* = 0.01), indicating that higher CS Index scores were associated with greater Stroop interference in the reading condition.

#### 3.2.1 Linear regression models

##### 3.2.1.1 Model 1: CS Index as a predictor

The CS Index significantly predicted *Stroop reading interference tendency* (*R* = 0.48, *R^2^* = 0.23, *p* = 0.01) (Table 7).

**Table 7:** CS Index as a predictor of Stroop reading interference tendency.

Model	Coefficient	Unstandardized	Standard error	Standardized	t	*p*
H_0_	(Intercept)	0.075	0.011		6.968	< 0.001
H_1_	(Intercept)	0.047	0.014		3.283	0.003
	CS-Index	9.174 × 10^−4^	3.538 × 10^−4^	0.476	2.593	0.016

#### 3.2.2 Multiple linear regression models

##### 3.2.2.1 Model 4: Stroop reading interference tendency

A multiple regression model incorporating the CS Index, AoA, education level and age accounted for a significant portion of the variance in Stroop reading interference tendency (*R* = 0.62, *R^2^* = 0.39, *p* = 0.036) (Table 8). The CS Index played a significant role in predicting Stroop reading interference tendency (*p* = 0.02). The other predictors did not show individual effects.

**Table 8:** CS Index, AoA, education level and age as predictors for Stroop reading interference tendency.

Model	Coefficient	Unstandardized	Standard error	Standardized	t	*p*
H_0_	(Intercept)	0.075	0.011		6.968	< 0.001
H_1_	(Intercept)	0.274	0.123		2.222	0.038
	CS-Index	9.142 × 10^−4^	3.650 × 10^−4^	0.474	2.504	0.021
	AoA	−0.007	0.005	−0.215	−1.212	0.239
	Education	−0.024	0.015	−0.324	−1.604	0.124
	Age	−0.004	0.003	−0.259	−1.289	0.212

## 4 Discussion

This study examined whether bilingualism lead to adaptations in EFs, and whether individual differences in habitual code-switching predict performance outcomes on EF tasks. To this end, first, Turkish-German bilinguals and German monolinguals were compared across five EF domains, response inhibition, working memory, task-switching, interference control, and attention. In addition, bilinguals’ naturalistic code-switching behavior was assessed using a novel quantitative index based on a scene description game. The findings revealed domain-specific group differences. Bilinguals outperformed monolinguals in the Task-Switching test, reflecting enhanced cognitive flexibility. However, monolinguals showed an advantage in working memory accuracy on the N-Back task. In the Stroop Interference test, bilinguals exhibited higher naming interference tendencies, indicating greater susceptibility to interference from incongruent stimuli. While bilinguals responded with higher accuracy on the Go/No-Go task, monolinguals had faster reaction times on the same task. The results of the attention-related tasks did not reveal significant differences between bilinguals and monolinguals. Importantly, habitual code-switching, as measured by the CS Index, emerged as a significant predictor of Stroop interference tendencies within bilinguals.

In line with our predictions, the findings support the notion that bilingualism impacts EF performance in a domain-specific manner Specifically, bilinguals demonstrated significant advantages in task-switching accuracy, consistent with prior research suggesting that managing two active linguistic systems enhances the ability to flexibly switch between tasks (Bialystok et al., 2012; Grundy, 2020). These findings also align with the ACH (Green and Abutalebi, 2013), which posits that bilinguals frequently engage cognitive control mechanisms such as interference suppression and task reconfiguration. However, contrary to expectations, bilinguals did not demonstrate superior response inhibition in the Go/No-Go task. Although they exhibited higher sensitivity index scores, they also had significantly slower reaction times. When reaction time was controlled for, the group difference in sensitivity index was no longer significant, suggesting that the bilinguals’ higher sensitivity may have been driven by a speed-accuracy trade-off rather than actual superior inhibitory control. In contrast, reaction time differences remained significant even after controlling for sensitivity scores, further indicating that bilinguals were generally slower in responding. Most studies on bilingual EF focus on speed rather than accuracy, and reported speed advantages often do not align with accuracy findings, suggesting a possible speed-accuracy trade-off (Struys et al., 2018). Notably, in a large meta-analysis of 152 studies on adults, Lehtonen et al. (2018) only included accuracy measures for response inhibition tasks, and found no bilingual advantage. In our study, the pattern of achieving accuracy at the cost of speed could reflect a response strategy shaped by bilingual experience in a predominantly monolingual society, where caution is emphasized to minimize errors. This cautious approach might manifest as careful decision-making on a stimulus-driven basis, adapting to the immediate demands of each trial. In the dual mechanisms of control model, Braver (2012) proposed that reactive control, which adapts based on task demands, often leads to slower but more accurate responses on incongruent trials. The findings in the present study can therefore be interpreted as bilinguals engaging reactive control in a way that favors accuracy, albeit at the expense of speed. Consistent with our predictions, the N-Back Task highlighted a relative disadvantage for bilinguals, as monolinguals demonstrated superior working memory accuracy, as measured by d-prime scores. This result is consistent with studies reporting no consistent bilingual advantage in working memory tasks (Ratiu and Azuma, 2015; Lukasik et al., 2018; Lehtonen et al., 2018; Nichols et al., 2020). The bilingual disadvantage observed in the N-Back task may be better understood as a trade-off. Working memory tasks such as the N-Back rely heavily on sustained proactive control, i.e., the continuous maintenance of task-relevant information (Braver, 2012). While some studies suggest that particular bilingual experiences—such as frequent use in dual-language contexts, high-entropy language environments, or structured alternational switching—foster proactive control (Hofweber et al., 2020; Green and Abutalebi, 2013; Gullifer and Titone, 2020), others highlight that bilinguals, particularly in dense code-switching contexts, tend to rely more on reactive control strategies (Green and Wei, 2014; Green, 2018; Hofweber et al., 2020; Beatty-Martínez et al., 2020). This interpretation aligns with the persistence–flexibility dilemma (Goschke, 2003), which describes the inherent trade-off between maintaining stability (persistence) and enabling adaptive shifts (flexibility). In our data, bilinguals showed enhanced task-switching accuracy, consistent with greater cognitive flexibility, but underperformed monolinguals on the N-Back, consistent with reduced persistence in sustained maintenance. These complementary findings suggest that bilingualism does not produce uniform advantages across EF domains, but rather shapes the balance between proactive and reactive control. Thus, the N-Back disadvantage in bilinguals may reflect a trait-level adaptation in cognitive control priorities, favoring flexibility at the cost of sustained maintenance. At the same time, we cannot exclude an alternative, more situational explanation. In this study, participants completed the scene description game immediately prior to the N-Back task. This dense code-switching context required continuous real-time processing of switches from their interlocutor and may have placed additional demands on cognitive resources. It is therefore possible that the observed bilingual disadvantage partly reflects these immediate interactional demands, rather than solely stable differences in working memory capacity. Notably, participants’ individual CS index did not predict N-Back performance, which could be consistent with this alternative explanation, although we emphasize that this interpretation remains tentative.

Contrary to our predictions, bilingual also exhibited less effective interference control as reflected in higher naming interference tendency in the Stroop task. One possible explanation lies in the preceding scene description game, whereby having been immersed in a dense code-switching context may have subsequently temporarily reduced the need for active interference control. Notably, the Stroop task was the only verbal EF task included in our study, leaving open the question of whether similar patterns would have emerged in the present sample in non-verbal tasks of interference control. While the Stroop task was deliberately chosen to reflect the bilingual experience of verbal interference, it is important to note that bilinguals generally perform more poorly than monolinguals in tasks requiring lexical access (Ivanova and Costa, 2008; Bialystok, 2009; Shook et al., 2015), pointing to the possibility that tasks with high verbal demands may inherently mask potential bilingual advantages in interference control.

Finally, as expected, there were no significant group differences in attention-related tasks, which primarily reflected basic attentional readiness and reaction speed, and imposed relatively low demands on cognitive control. Bialystok and Craik (2022) suggest that bilinguals are expected to outperform monolinguals to the extent that the attentional control demands of the task exceed the abilities of monolinguals but not bilinguals. Thus, it is expected that no group differences emerge on tasks that can be performed in an automated manner, or are within the attentional control capabilities of the population, such as simple EF tasks performed by young adults.

Together, these findings further emphasize that bilingual adaptations do not generalize uniformly across all EF domains but are contingent on the specific cognitive demands of the task (Lowe et al., 2021). The differential performance patterns across tasks can also be understood through the lens of the persistence-flexibility dilemma (Goschke, 2003), which describes the competing demands on cognitive control to balance stability (persistence) and adaptability (flexibility). Bilinguals, by necessity, develop strategies that prioritize flexibility to manage language switching and interference, which may explain their superior task-switching performance. However, this same emphasis on flexibility may come at the cost of persistence-related processes, such as the stable maintenance of information required in working memory tasks. This trade-off underscores the interplay between persistence and flexibility within EF domains and helps contextualize the domain-specific effects observed in bilinguals. This trade-off also reflects the dual nature of bilingual cognitive control, where flexibility enables adaptive performance in dynamic contexts, while persistence supports tasks requiring prolonged suppression of competing stimuli.

Within the bilingual group, in line with our expectations, habitual code-switching behavior predicted poorer interference control. High levels of dense code-switching as reflected in CS index scores were associated with greater interference in the reading condition. Our finding that higher code-switching frequency predicted greater susceptibility to Stroop interference is consistent with theoretical accounts proposing that dense code-switching induces a broad attentional state (Green, 2018). While such a state supports flexibility and rapid reconfiguration across languages, it may reduce the ability to maintain selective focus, thereby increasing vulnerability to interference under conditions of high cognitive demand. This pattern illustrates a domain-specific trade-off: dense code-switching may foster adaptive flexibility in everyday bilingual communication, but at the expense of persistence-related processes such as interference suppression in Stroop tasks. This interpretation aligns with frameworks emphasizing the balance between proactive and reactive control and the persistence–flexibility dilemma (Goschke, 2003; Braver, 2012). While the Stroop task is well-suited for examining interference suppression, it may also reveal the cognitive costs associated with managing multiple linguistic systems in highly automatic processes such as reading. In a verbal paradigm conducted in German, such costs could be modulated by participants’ relative language dominance. Bilinguals with stronger Turkish dominance, for instance, might experience heightened lexical competition when processing German stimuli, thereby amplifying interference effects. Although all bilingual participants in this study were highly proficient in German, we did not include language dominance in our assessment, and variability in dominance remains a potential contributing factor. This constitutes a limitation of the present design and reduces the generalizability of our findings, as bilinguals dominant in the non-testing language may show different interference patterns. Future research should therefore examine both verbal and nonverbal Stroop tasks across bilinguals with varying dominance profiles.

In contrast to our predictions, CS index did not predict individual task-switching performance within the bilingual group, although bilinguals outperformed monolinguals overall. This suggests that in our sample the general bilingual experience, rather than the specific habitual code-switching patterns, accounted for enhanced cognitive flexibility.

Within the bilingual group, an additional finding emerged with regards to AoA. The AoA of German moderately correlated with reaction times in the Go/No-Go task, with a later AoA associated with slower reaction times. Few studies have explored the relationship between AoA and EF performance, yielding mixed results across different domains (Luk et al., 2011; Tao et al., 2011; Pelham and Abrams, 2014). While some evidence points to benefits of an earlier AoA, most studies categorize bilinguals into groups of early and late bilinguals, with a typical cutoff age around 7 or later. In our study, all bilingual participants were early bilinguals who either acquired German alongside Turkish from infancy or began learning German by the age of 6 at the latest. This finding suggests that even within an early bilingual group seemingly balanced in AoA, subtle variations in the timing of second language acquisition can influence EF performance. This aligns with recent studies that show that even small differences in AoA can have significant cognitive effects, even in populations considered to be “early bilinguals” (Soveri et al., 2011; Yow and Li, 2015).

Overall, these results illustrate the complex interplay between bilingualism, code-switching behavior, and EFs. They reinforce the domain-specific nature of bilingual cognitive adaptations and emphasize the importance of considering individual differences in bilingual experiences, such as code-switching habits and context. The findings also highlight the need for future research to explore the cognitive mechanisms underlying different types of code-switching (e.g., insertions, alternations, or dense switching) and their specific impacts on components of EF. Moreover, these findings contribute to theoretical frameworks like the ACH (Green and Abutalebi, 2013) and the persistence-flexibility dilemma (Goschke, 2003), offering empirical support for the idea that bilingualism involves a dynamic trade-off between stability and adaptability in cognitive control. Understanding how these processes interact across tasks and domains will provide deeper insights into the effects of bilingualism on cognition.

This study has several limitations that should be considered when interpreting the findings. The relatively small sample size, particularly within the bilingual group, limits statistical power and generalizability. As such, larger and more diverse samples are necessary to confirm these findings and explore potential subgroup differences, such as those based on age of acquisition, language dominance, and proficiency. In our study, we focused on early bilingualism to establish a clear contrast between the groups. However, it is important to note that all participants, including those classified as monolinguals, reported at least some proficiency in another language, most commonly English. Future studies may therefore benefit from more fine-grained assessments of participants’ broader linguistic repertoire. Furthermore, the exclusive focus on Turkish-German bilinguals may limit generalizability, as language distance and structural differences can uniquely influence cognitive demands. Future research efforts may consider examining whether similar patterns emerge in bilingual speakers of both structurally distinct and typologically similar language pairs.

A considerable limitation of this study is the use of a single task to represent each EF construct in question (e.g., one task for response inhibition, one for task-switching). It is well-established that any individual EF task is multifactorial, capturing not only the core construct of interest but also task-specific variance, often termed “task impurity” (Miyake et al., 2000). As such, even within each EF domain, different tasks may engage distinct sub-processes, thereby limiting the extent to which task-specific findings can inform domain-level conclusions (Ware et al., 2020). While multivariate approaches such as Principal Component Analysis (PCA) or Structural Equation Modeling (SEM) could potentially address this by extracting latent variables, our data and overall sample size did not meet the necessary statistical prerequisites for achieving stable solutions in either PCA or SEM (Comrey and Lee, 1992). Consequently, our conclusions are appropriately interpreted with caution, relating specifically to the operationalization of the EF components as measured by our selected tests (e.g., “task-switching as measured by the alternating-runs paradigm”). Future research with larger samples and a broader range of tasks per construct is needed to successfully model these abilities as latent factors and provide a clearer picture of bilingual effects on EFs.

A further methodological limitation concerns the reliability of individual-differences measures. In particular, difference scores such as Stroop interference are known to suffer from reduced reliability (Hedge et al., 2018). While our study employed standardized tasks from the Vienna Test System with established psychometric properties, we acknowledge that this limitation applies to the interpretation of difference scores in our analyses, and therefore interpret these results with appropriate caution.

The aggregated nature of our CS index further constraints our investigation. Although this approach successfully quantified participants’ overall propensity to code-switching and provided an ecologically valid, data-driven alternative to self-report measures, it did not allow us to differentiate between specific switch types (e.g., insertions, alternations, dense switching). This is an important issue, as theoretical frameworks have argued that different types of code-switching are associated with distinct cognitive control processes (Green and Abutalebi, 2013; Green and Wei, 2014; Green, 2018). Due to the relatively low frequency of individual switch types in our modest sample, separate analyses would have yielded unreliable estimates, and a categorical classification into low and high switchers would have carried the same risk. Our methodological decision to collapse across types was therefore motivated by the need to balance ecological validity and statistical robustness. Nevertheless, we acknowledge that future studies with larger bilingual cohorts are crucial to quantify different code-switching types independently and investigate their specific predictive power for distinct EF domains. Moreover, in addition to code-switching types, the CS index presented in this study can be used in future studies to distinguish between participant-initiated switches and those triggered by the confederate’s switch, allowing for the isolation of the interlocutor’s influence.

Furthermore, the study’s cross-sectional design precludes causal interpretations of the relationship between code-switching behavior and EF. Longitudinal research is necessary to determine whether habitual code-switching enhances specific EFs over time or whether pre-existing cognitive differences s influence code-switching behavior. A dynamic bidirectional relationship is conceivable, where cognitive abilities and language-switching practices mutually influence and reinforce one another, creating a feedback loop over time. In the present study, participants had the freedom to decide whether to engage in code-switching during the scene description game. As such, opting to switch languages rather than sticking to one in this context may have reflected higher levels of cognitive flexibility. This bidirectional interaction could mean that individuals with heightened cognitive abilities in certain domains may engage in more frequent or complex code-switching, which in turn further refines their EFs. Future studies should explore this possibility to provide a more comprehensive understanding of the interplay between bilingual language use and cognitive processes. Moreover, the scene description game, while innovative in its naturalistic approach, might not fully represent participants’ real-world code-switching behavior due to its structured format. Although the confederate was a Turkish-German bilingual, simulating authentic bilingual interactions and promoting the natural use of both languages, the scripted nature of the game may have constrained some aspects of spontaneous language use. Future studies could benefit from incorporating a broader range of interlocutors and scenarios to better capture the dynamic and context-sensitive nature of code-switching behavior and its cognitive effects. Finally, a wider variety of EF tasks that differ in modality, domain, and complexity could help disentangle the domain-specific and domain-general effects of bilingualism and code-switching on EF.

## Data Availability

The raw data supporting the conclusions of this article will be made available by the authors, without undue reservation.

## 1

Dieck, L., Dirker, D., and von Stutterheim, C. (2025). A comprehensive approach for assessing code-switching behaviour. [Manuscript submitted for publication].
