# Correction: The impact of bilingualism and code-switching on executive function performance

**DOI:** 10.3389/fpsyg.2026.1824496

**Published:** 2026-04-20

**Authors:** Basak Özkara, Raul Schneider, Gülay Cedden, Christiane von Stutterheim, Patric Meyer

**Affiliations:** 1 Institute of Psychology, Heidelberg University, Heidelberg, Germany; 2 School of Psychology, SRH University Heidelberg, Heidelberg, Germany; 3 Institute of German as a Foreign Language Philology, Heidelberg University, Heidelberg, Germany; 4 EEG and Language Processing Laboratory, Faculty of Education, Middle East Technical University, Ankara, Türkiye

**Keywords:** bilingualism, code-switching, executive functions, cognitive control, bilingual advantage

In the original version of this article, the correlations involving age of acquisition (AoA) and education level reported in Section 3.2 (Bilingual within-subject analyses) emerged as a result of incorrect data filtering. After correcting this error and re-running the analyses, these associations no longer met the predefined inclusion criterion (*p* < 0.10) for subsequent regression analyses. Consequently, the regression models that were conducted based on these preliminary associations are no longer warranted under the stated analytic procedure.

A correction has been made to the Section 3.2 Bilingual within-subject analyses, Paragraph 1:

The following text has been removed:

“The analysis revealed two significant correlations between key variables and EF performance measures. Additionally, AoA was positively correlated with reaction times in response inhibition (*r* = 0.284, *p* = 0.02), suggesting that earlier language acquisition was linked to faster inhibitory responses. A trend toward a negative correlation was observed between education and Stroop naming interference tendency (*r* = −0.241, *p* = 0.07). Although this relationship did not reach statistical significance, it was further investigated with linear regression analyses along with the significant findings.”

The paragraph now reads:

“The analysis revealed a significant positive correlation between the CS Index and Stroop reading interference tendency (*r* = 0.476, *p* = 0.01), indicating that higher CS Index scores were associated with greater Stroop interference in the reading condition.”

A correction has been made to the sections 3.2.1 Linear regression models and 3.2.2 Multiple linear regression models:

Because AoA and education level no longer met the predefined correlation threshold for inclusion in regression analyses, the following models which were conducted based on those preliminary associations have been removed:

- 3.2.1.2 Model 2: Education level as a predictor
- 3.2.1.3 Model 3: AoA as a predictor
- 3.2.2.2 Model 5: Stroop naming interference tendency
- 3.2.2.3 Model 6: Response inhibition reaction time

Accordingly, Tables 8, 9, 11, and 12 have been removed.

A correction has been made to Section 4 Discussion, Paragraph 8:

This paragraph has been removed, as the underlying association between AoA and response inhibition reaction time is no longer statistically significant following correction of the data filtering error, and the corresponding interpretation is therefore not supported.

The corresponding author's email address in the published article is incorrect. The correct contact email is basakozkara@gmail.com.

The original version of this article has been updated.

