# Supplementary material for: The impact of bilingualism and code-switching on executive function performance
Source: Front Psychol. 2025 Dec 3;16:1583441. doi: 10.3389/fpsyg.2025.1583441 (PMC12709169; doi:10.3389/fpsyg.2025.1583441)
Supplement: Supplementary file 1 [file Table_1.docx]

**Supplementary Material**

Means and standard deviations for each group and EF outcome variable

| **EF Task** | **Outcome variables** | **Monolingual M (SD)** | **Bilingual M (SD)** |
| --- | --- | --- | --- |
| Go/No-Go Task | Sensitivity Index | 3.17 (0.61) | 3.50 (0.61) |
|  | Reaction Time | 0.26 (0.02) | 0.28 (0.04) |
| N-Back Task | Number of correct trials | 9.45 (2.89) | 8.29 (2.91) |
|  | Number of error trials | 5.78 (3.52) | 7.46 (4.08) |
|  | Number of omission trials | 4.55 (2.89) | 5.67 (2.95) |
|  | Reaction Time Correct Trials | 0.72 (0.16) | 0.75 (0.15) |
|  | Reaction Time Errors | 0.89 (0.17) | 0.86 (0.14) |
|  | d-prime | 3.44 (4.22) | 0.82 (5.44) |
| STROOP Task | Reaction Time Reading (Congruent) | 0.81 (0.14) | 0.77 (0.10) |
|  | Reaction Time Reading (Incongruent) | 0.87 (0.13) | 0.84 (0.10) |
|  | Reaction Time Naming (Congruent) | 0.68 (0.08) | 0.67 (0.08) |
|  | Reaction Time Naming (Incongruent) | 0.75 (0.10) | 0.76 (0.08) |
|  | Reading Interference Tendency | 0.07 (0.04) | 0.07 (0.05) |
|  | Naming Interference Tendency | 0.05 (0.03) | 0.07 (0.03) |
| Task-Switching Task | Accuracy | 1.97 (4.30) | 4.14 (4.21) |
|  | Speed | 0.24 (0.14) | 0.22 (0.16) |
| Intrinsic Alertness Task | Intrinsic alertness (visual) | 198.39 (16.68) | 201.29 (17.31) |
| Cross-Modal Divided Attention Task | Cross-modal divided attention (visual/auditory) | 366.19 (60.70) | 358.15 (50.57) |
